# Correlation between magnifying narrow band imaging and histopathology in gastric protruding/or polypoid lesions: a pilot feasibility trial

**DOI:** 10.1186/1471-230X-12-17

**Published:** 2012-02-22

**Authors:** Takafumi Omori, Yoshio Kamiya, Tomomitsu Tahara, Tomoyuki Shibata, Masakatsu Nakamura, Joh Yonemura, Masaaki Okubo, Daisuke Yoshioka, Takamitsu Ishizuka, Naoko Maruyama, Toshiaki Kamano, Hiroshi Fujita, Yoshihito Nakagawa, Mitsuo Nagasaka, Masami Iwata, Tomiyasu Arisawa, Ichiro Hirata

**Affiliations:** 1 Department of Gastroenterology, Fujita Health University School of Medicine, 1-98 Dengakugakubo, Kutsukake-cho, Toyoake, Aichi 470-1192, Japan; 2 Department of Gastroenterology, Kanazawa Medical University, 1-1 Daigaku, Uchinadamachi, Ishikawa 920-0293, Japan

**Keywords:** NBI, Stomach, Magnifying endoscopy, Polypoid lesions, Gastric cancer

## Abstract

**Background:**

Several study showed usefulness of microscopic capillaries, seen by magnifying narrow band imaging (NBI) endoscopy for predicting histopathology among superficial depressed or flat elevated gastric neoplasia (GN). Here we assessed the diagnostic efficacy of magnifying NBI for predicting histopathology among gastric protruding/or polypoid lesions.

**Methods:**

Using endoscopic pictures of magnifying NBI from 95 protruding/or polypoid lesions (19 fundic gland polyps: FGP, 47 hyperplastic polyps: HP, and 29 GN), fine mucosal patterns were classified into four categories: small round, prolonged, villous or ridge, and unclear patterns, and micro vascular patterns were classified into five categories: honey comb, dense vascular, fine net work, core vascular, and unclear patterns.

**Results:**

Most suggestive micro vascular patterns for predicting FGP, and HP were honeycomb (sensitivity 94.7%, specificity 97.4%), and dense vascular patterns (sensitivity 93.6%, specificity 91.6%), respectively. Fine net work, core vascular, and unclear patterns presented higher specificity (97%, 100%, and 100%) for predicting GN, and diagnostic efficacy of combined of those patterns was favorable (sensitivity 86.2%, specificity 97.0%).

**Conclusion:**

Micro vascular patterns by using magnifying NBI provides meaningful information for predicting the histopathology of gastric protruding/or polypoid lesions.

## Background

The narrow-band imaging (NBI) system is an endoscopic imaging technique for the enhanced visualization of mucosal microscopic structure and capillaries of the superficial mucosal layer. Images are obtained by using two narrow wave bands of light (blue: 390-445 nm; green: 530-550 nm), which are different from conventional red-green-blue filters. These two narrow wave bands are easily absorbed by hemoglobin in the blood [1]. Combining the NBI system and magnifying endoscopy allows for simple and clear visualization of microscopic structures of the superficial mucosa and its capillary patterns. Preliminary studies using NBI for identifying intestinal metaplasia and adenocarcinoma in the esophagus, [2,3] squamous dysplasia in the bronchi, [4] and mucosal patterns in colon polyps [5] have suggested its potential usefulness.

In the stomach, it has also been reported that the mucosal appearance seen by magnifying NBI endoscopy is useful for diagnosing gastric intestinal metaplasia, and histological severity of gastritis [6-8].

Concerning the potential usefulness of magnifying NBI endoscopy for diagnosing gastric neoplastic lesions, several study showed the potential usefulness of microscopic capillaries, seen by magnifying NBI for predicting gastric neoplasia among superficial depressed [9], or flat elevated early gastric neoplastic lesions [10]. However, there was no report showing its efficacy for discriminating the histopathology of gastric protruding/or polypoid lesions.

Consequently, this study was designed to investigate the prevalence of fine mucosal patterns and micro vascular patterns, seen by using magnifying NBI endoscopy in gastric protruding/or polypoid lesions and its relation to the histopathology.

## Methods

### Patients

Total of 95 protruding/or polypoid lesions from 76 consecutive individuals who received upper gastroscopy concurrently with conventional white light (WL) upper gastroscopy and magnifying NBI at the endoscopy Center of Fujita Health University, were considered eligible for enrolment. All 76 individuals were diagnosed as having protruding/or polypoid lesions in their stomachs. The histopathological diagnosis of protruding/or polypoid lesions were, 19 fundic gland polyps (FGP) from 17 individuals, 47 hyper plastic polyps (HP) from 30 individuals, and 29 gastric neoplasia (GN) from 29 individuals. The 29 GN consisted of 27 well differentiated adenocarcinomas, and 2 gastric adenomas. Histopathological analysis also confirmed that all GN were early stage in which tumor invasion is confined to the mucosa or submucosa. All histopathological diagnosis was performed by pathologist in our hospital. 29 individuals with GN were admitted to our hospital for laparoscopic surgery or for endoscopic mucosal dissection. Other participants underwent upper gastroscopy for various indications, including yearly screening for gastric cancer, secondary complete check-up after barium radiographic examination due to a suspicion of gastric cancer or polyps, and complaints of abdominal discomfort. Fujita Health University School of Medicine approved the study protocol, and informed consent was obtained from all participants.

### Endoscopic procedure

Before endoscopic examination was begun, 20,000 U of Pronase (Pronase MS; Kaken Pharmaceutical Products Inc, Tokyo, Japan) was administered to each participant to remove gastric mucus. Upper gastroscopy was performed with an Olympus GIF-H260Z (Olympus Medical Systems Co, Tokyo, Japan) by seven experienced endoscopists (M.N, Y.K, T.T, M.W, T.S, M.O, and Y,N); each of them had previously carried out a minimum of 2500 upper gastroscopies. Endoscopic pictures for each lesion were taken in the order of conventional white light endoscopy (WL) at first, then the NBI light source was turned on, and the surface patterns of each lesion were carefully evaluated with high power magnification (x 85), the distal tip of the endoscope being attached to the mucosa. During the procedure, a transparent attachment was attached approximately 3.0 mm distal to the tip of the endoscope to maintain the focal distance. When the specific fine mucosal structures, or micro vascular patterns were identified, the most predominant appearance was scanned, endoscopic photographs were taken. For all 29 GN, laparoscopic surgery or endoscopic mucosal dissection was performed, while biopsy, polypectomy or endoscopic mucosal resection was performed for the histopathological assessment of other non-neoplastic lesions. For the non-neoplastic lesions more than 1 cm in size, at least 2 biopsies, polypectomy or endoscopic mucosal resection was performed to confirm that the lesion is non-neoplastic.

### Image evaluation, and classification of fine mucosal structures and micro vascular patterns seen by magnifying NBI system

**Figure 1:**
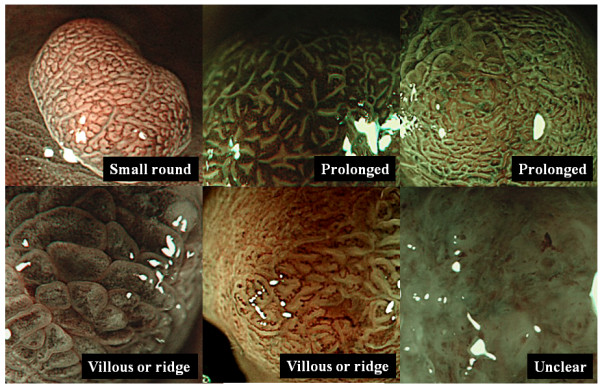
**Classification of fine mucosal structures of gastric polypoid lesions**. Fine mucosal structures were classified into following four categories: small round (uniformly arranged small round pits), prolonged (long holed or oval shaped pits), villous or ridge (villous or ridge like structures, pit is not seen), and unclear (structures is not observed) patterns.

**Figure 2:**
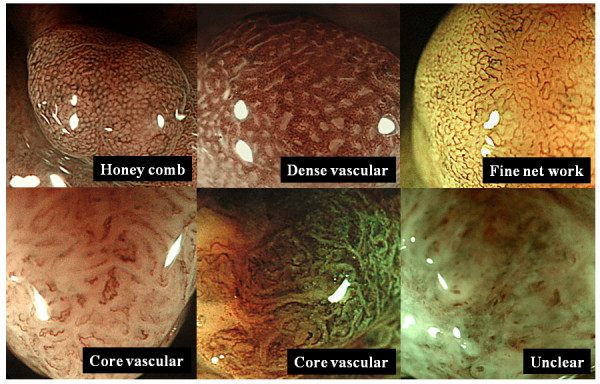
**Classification of capillary patterns of gastric polypoid lesions**. The micro vascular patterns were classified into following five categories: honey comb (uniformly arranged honey comb like appearance), dense vascular (increased density of irregular vessels in the most of area of the micro structure), fine net work (net like appearance consisted of irregular shaped micro vessels), core vascular (clearly visible coiled or wavy vessels in the central area of the mucosal structure), and unclear patterns (micro vascular patterns is not observed).

Most typical two or three pictures, showing the characteristic of each lesion was selected from both taken by WL and magnifying NBI. By using pictures of WL, location of lesions was defined according to anatomical distribution. Lesions were also classified macroscopically according to Yamada's classification [11]. Yamada type I polyps are elevated, with an indistinct border. Type II polyps are elevated with a distinct border at the base but no notch. Type III polyps are elevated, but no peduncle. Type IV polyps are pedunculated and elevated. In addition, size, color and the presence of uneven form or depressed area were evaluated. By using pictures taken by using magnifying NBI, fine mucosal structures of the 95 lesions were classified into following four categories: small round (uniformly arranged small round pits), prolonged (long holed or oval shaped pits), villous or ridge (villous or ridge like structures, pit is not seen), and unclear (structures is not observed) patterns (Figure 1). Also, the micro vascular patterns were classified into following five categories: honey comb (uniformly arranged honey comb like appearance), dense vascular (increased density of irregular vessels in the most of area of the micro structure), fine net work (net like appearance consisted of irregular shaped micro vessels), which has been reported to be seen in differentiated depressed gastric cancer (9), core vascular (clearly visible coiled or wavy vessels in the central area of the mucosal structure), and unclear patterns (micro vascular patterns is not observed) (Figure 2). All endoscopic pictures were sorted by three well experienced endoscopists who were blinded to histological data (T.T, Y.K, and T.O), and diagnosis of WL endoscopy and classification of magnifying NBI imagings were performed by consensus manner with the same members.

### Statistical analysis

The association between gender, Yamada's classification, size, color and the presence of uneven form or depressed area, location of lesions, and histopatological diagnosis of lesions were assessed by the Chi-squire test with Yates' continuity correction. The association between age and histopatological diagnosis of lesions were assessed by the One-way ANOVA. A probability value of less than 0.05 was considered statistically significant in above analysis. Diagnostic efficacy of fine mucosal structures and micro vascular patterns seen by magnifying NBI for predicting histopathology gastric lesions was evaluated in terms of sensitivity and specificity. For the assessment of interobserver accordance, κ values was evaluated. A k value below 0.4 was regarded as representing poor agreement, a k value of 0.41-0.60 fair agreement, a k value of 0.61-0.80 good agreement, and a k value greater than 0.80 excellent agreement.

## Results

### Study population, and macroscopic appearance of gastric protruding/or polypoid gastric lesions

Clinical data of 95 gastric protruding/or polypoid lesions from 76 individuals are shown in the Table 1.

**Table 1:** Clinicopathological characteristics of subjects

	Histopathological diagnosis			*p *value	
	
Variables	FGP	HP	GN	GN *vs*.FGP	GN *vs*. HP
Number of patients	17	30	29	ND	ND
Number of pathological lesions	19	47	29	ND	ND
Gender (male/female)	9(52.9)/8(47.1)	16(53.3)/14(47.7)	23(79.3)/6(20.7)	NS	NS
Age (mean ± SD)	54.9 ± 23.6	67.5 ± 9.4	72.5 ± 6.9	< 0.0001	0.03
Yamada's classification					
*I and II*	8(42.1)	15(31.9)	2(6.9)	0.04	NS
*III*	10(52.6)	22(46.8)	19(65.5)	NS	NS
*IV*	1(5.3)	10(21.3)	8(27.6)	NS	NS
Size					
*< 5 mm*	7(36.8)	17(36.2)	0(0)	0.002	0.0007
*5 mm ~ < 10 mm*	10(52.6)	16(34.0)	12(41.4)	NS	NS
*10 mm ~ < 20 mm*	2(10.5)	8(17.0)	9(31.0)	NS	NS
*20 mm ~*	0(0)	6(12.8)	8(27.6)	0.03	NS
Color					
*Whitish*	4(21.1)	0(0)	5(17.2)	NS	0.01
*Same as surrounded mucosa*	15(78.9)	2(4.3)	9(31.0)	0.003	0.004
*Reddish*	0(0)	45(95.7)	15(51.7)	0.0005	0.00002
Location					
*Upper third*	8(42.1)	7(42.1)	6(20.7)	NS	NS
*Middle third*	11(57.9)	17(36.2)	7(24.1)	0.04	NS
*Lower third*	0(0)	23(48.9)	16(55.2)	0.0003	NS
Uneven form or presence of depressed area	2(10.5)	8(14.2)	12(41.4)	0..48	0.04
Erosion	0(0)	12(25.5)	8(27.6)	0.03	NS

FGP, funding grand polyps; HP hyperplastic polyps; Gn, gastric neoplasia; ND not done

GN consisted of 27(93.1%) well differentiated adeno carcinomas, and 2(6.9%) gastric adenomas

All *p *values were assessed by the Chi-square test, with Yates' continuity correction except for the association of age

Age was assessed by the One-way ANOVA

Patients with GN presented higher age, lower prominence of Yamada's type I and II compared to FGP, and lower prominence of less than 5 mm, and higher prominence of 20 mm or higher in size.

Additionally, colors, locations, and presence of depressed area, or lesions showed several significant p values when comparing GN with FGP or HP.

### Diagnostic efficacy of fine mucosal structures and micro vascular patterns seen by magnifying NBI for predicting histopathology of gastric protruding/or polypoid gastric lesions

Prevalence of fine mucosal structures and micro vascular patterns, and combined of both patterns in 95 gastric protruding/or polypoid lesions, and sensitivity and specificity for predicting GN are shown in Table 2. Concerning the fine mucosal structures, small round pattern seemed to be a suggestive pattern for predicting FGP (sensitivity 94.7%, specificity 93.4% for predicting FGP), and unclear pattern seemed to be a specific pattern of GN (sensitivity 17.2%, specificity 100% for predicting GN). However, the sensitivity of unclear pattern for predicting GN was low, due to the small prevalence of this pattern in GN cases (five out of 29 cases). Also, sensitivity and specificity of other patterns were not capable of distinguishing GN and HP. However, it was revealed that the micro vascular patterns had a good correlation with histopathology of gastric lesions. Most suggestive patterns for predicting FGP, and HP were combined of honeycomb (sensitivity 94.7%, specificity 97.4%), and dense vascular patterns (sensitivity 93.6%, specificity 91.6%), respectively. Fine net work, core vascular, and unclear patterns showed higher specificity (97%, 100%, and 100%) for predicting GN, and diagnostic efficacy of combined of those capillary patterns was relatively favorable (sensitivity 86.2%, specificity 97.0%). We also examined diagnostic efficacy of combined of both fine mucosal structures and micro vascular patterns, but diagnostic efficacy was not improved, compared to micro vascular patterns it self. Most suggestive patterns for predicting fundic grand polyps, and hyperplastic polyps were combined of small round/honeycomb (sensitivity 94.7%, specificity 97.4%), and prolonged and villous or ridge/dense vascular patterns (sensitivity 93.6%, specificity 91.6%), respectively. Most favorable combined patterns for predicting GN was small round/fine net work+ prolonged/core vascular+ villous or ridge/core vascular+ unclear/core vascular+ unclear/unclear patterns (sensitivity 86.2%, specificity 97.0%).

**Table 2:** Prevalence of fine mucosal structures and micro vascular patterns seen by magnying NBI, among gastric protruding/or polypoid lesion, and diagnostic efficacy of those patterns for GN

	Histopathological diagnosis			Diagnostic efficacy for GN	
	
	FGP	HP	GN	Sensitivity (%)	Specificity (%)
Fine mucosal structures					
*Small round*	18(94.7)	2(4.3)	3(10.3)	10.3	69.7
*Prolonged*	0(0)	15(31.9)	1(3.5)	3.5	77.3
*Villous or ridge*	1(5.3)	30(63.8)	20.(69.0)	69.0	53.0
*Unclear*	0(0)	0(0)	5(17.2)	17.2	100
Micro vascular patterns					
*Honey comb*	18(94.7)	2(4.3)	0(0)	0	69.7
*Dense vascular*	0(0)	44(93.6)	4(13.8)	13.8	33.3
*Core vascular*	1(5.3)	1(2.1)	21(72.4)	72.4	97.0
*Fine net work*	0(0)	0(0)	3(10.3)	10.3	100
*Unclear*	0(0)	0(0)	1(3.5)	3.5	100
Combined of fine mucosal structures and micro vascular patterns					
*Small round/Honey comb*	18(94.7)	2(4.3)	0(0)	0	69.7
*Prolonged/Dense vascular*	0(0)	14(29.8)	0(0)	0	78.8
*Villous or ridge/Dense vascular*	0(0)	30(63.8)	4(13.8)	13.8	54.5
*Small round/Fine net work*	0(0)	0(0)	3(10.3)	10.3	100
*Prolonged/Core vascular*	0(0)	0(0)	1(3.5)	3.5	100
*Villous or ridge/Core vascular*	1(5.3)	1(2.1)	16(55.2)	55.2	97.0
*Unclear/Core vascular*	0(0)	0(0)	4(13.8)	13.8	100
*Unclear/Unclear*	0(0)	0(0)	1(3.5)	3.5	100

FGP fundic grand polyps; HP, hyperplastic polyps; GN, gastric neoplasia; ND, not done

Most favourable combination of micro vascular pattern for diagnosing GN were; Core vascular+ fine network+ unclear: sensitivity 86.2%, specificity 97.0%

Most favourable combination of fine mucosal structures and micro vascular pattern for diagnosing GN were; small round/fine net work+ prolonged/core vascular+ villous or ridge/core vascular+ unclear/core vascular+ unclear: sensitivity 86.2%, specificity 9.0%

### Histological findings of four cases of GN, presented with dense vascular patterns

**Figure 3:**
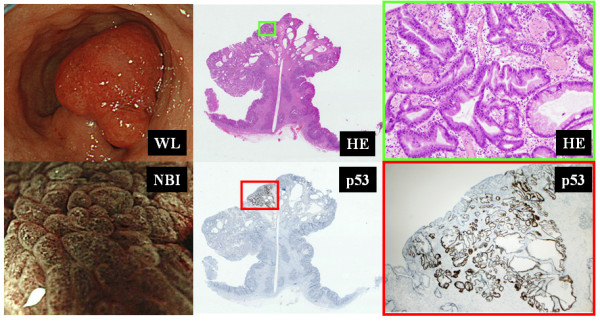
**A case of gastric adenocarcinoma, presented with villous or ridge mucosal structure and dense vascular pattern**. Histopathological finding of this cases showed small component of well differentiated adenocarcinoma in hyperplastic polyp (green frame), consisted with p53 immunostaining (red frame). WL, white light endoscopy; NBI, narrow band imaging; HE, hematoxylin-eosin staining.

Although, dense vascular pattern seemed to be a suggestive pattern for predicting HP, it actually contained four cases of GN (Table 2). Therefore, we reviewed histopathological findings of those cases. Fine mucosal structures of all four cases was villous or ridge patterns (Table 2). All were well differentiated adenocarcinomas. A case of gastric adenocarcinoma, presented with villous or ridgemucosal structure and dense vascular pattern was shown in Figure 3. Histopathological finding of this cases showed small component of well differentiated adenocarcinoma in hyperplastic polyp, consisted with p53 immunostaining. Other three cases with villous or ridge/dense vascular patterns also showed the focal components of well differentiated adenocarcinomas in hyperplastic polyps in various degrees (data not shown).

### Interobserver concordance regarding micro vascular patterns

Since it was suggested that the micro vascular patterns had a good correlation with histopathology of gastric lesions, we analyzed interobserver concordance using κ coefficient values. For this analysis, three additional experienced endoscopists who were blinded to histological diagnosis participated. We first explained to them about the classification of micro vascular patterns by using typical endoscopic picture of two cases for five micro vascular patterns. Then we assessed κ coefficient values using endoscopic pictures of remaining cases.

Regarding the four micro vascular patterns (honey comb, dense vascular, fine net work and core vascular) κ values were good ranging from 0.64 to 0.75. The value of unclear pattern was excellent (0.89). (Table 3)

**Table 3:** Interobserver concordance (κ coefficient values) for micro vascular patterns

Micro vascular patterns	κ values
*Honey comb*	0.75
*Dense vascular*	0.73
*Fine net work*	0.64
*Core vascular*	0.73
*unclear*	0.89

## Discussion

So far, several study showed the potential usefulness of microscopic capillaries, seen by magnifying NBI for predicting gastric neoplasia among superficial depressed or flat elevated early gastric neoplastic lesions [9,10]. However, there was no report showing its efficacy in gastric protruding/or polypoid lesions.

In this study, fine mucosal patterns and capillary patterns of the gasric protruding/or polypoid lesions could be classified into four and five categories, respectively. In particular, micro vascular patterns had good correlations with histopathology. Honey comb, and dense vascular patterns showed higher sensitivity and specificity for predicting FGP (sensitivity 94.7%, specificity 97.4%), and HP (sensitivity 93.6%, specificity 91.6%) respectively. FGP is almost always associated with non inflamed normal gastric mucosa, showing hyperplasia of non neoplastic gastric fundic grand [12], while HP were usually associated with *H. pylori *related chronic gastritis, characterized by dilated, tortuous gastric foveoli set within an inflamed, edematous stroma [13]. Therefore, it may be reasonable to speculate that hyperplasia of normal gastric fundic grand may reflects uniformly arranged honey comb like appearance of the FGP, which is often observed in normal gastric mucosa in the corpus [7,8], and the continuous destruction and regeneration of new vessels and edema due to severe inflammation may reflect increased density of irregular vessels in the most of area of the micro structure, seen in HP.

On the other hand, fine net work, core vascular, and unclear patterns presented higher specificity (97%, 100%, and 100%) for predicting GN, and diagnostic efficacy of combined of those micro vascular patterns was favorable (sensitivity 86.2%, specificity 97.0%). Although we could predict the GN from the information of conventional endoscopic findings (anatomical location, Yamada's classification, size, color and the presence of Uneven form or presence of depressed area), it was clear that diagnostic efficacy of micro vascular pattern was better, suggesting that micro vascular patterns by magnifying NBI may grow to be a promising tool for diagnosing gastric protruding/or polypoid lesions. We also examined diagnostic efficacy of combined of both fine mucosal structures and micro vascular patterns, but diagnostic efficacy was not improved compared to micro vascular patterns it self. Our result suggests that, in the gastric protruding/or polypoid lesions, combined of microvascular patterns, rather than fine mucosal patterns are most useful sign for discriminating GN from FGP and FP. Although GN in our study were histologically well differentiated adenocarcinomas or gastric adenomas, and they did not contained undifferentiated carcinoma components (data not shown), in addition to the fine net work pattern, often seen in well differentiated superficial depressed adenocarcinomas [9], protruding/or polypoid GN showed at least two more suggestive micro vascular patterns: core vascular, and unclear patterns. This is possibly due to feature of gastric protruding/or polypoid lesions. Also, when considering all histological types including FGP and HP, we needed at least five micro vascular patterns to distinguish all lesions. Although our result indicated that five micro vascular patterns had good interobserver concordance in experienced endoscopists, whether our criteria could be well accepted for all of endoscopist, including trainee of magnifying NBI, need to be validated.

In our study, dense vascular pattern, which is a suggestive pattern for HP actually contained four cases of well differentiated adenocarcinomas. Histological examination revealed that the focal components of well differentiated adenocarcinomas in hyperplastic polyps in various degrees, suggesting that this pattern may have possibility of containing small, but considerable percentage of neoplastic components. Although the HP itself are non-neoplastic, dysplastic changes and/or gastric adenocarcinoma may develop within the lesion in rare [14]. However, the risk of developing an adenocarcinoma within a hyperplastic polyp has been estimated from 0 to 8% (mean 2.1%) and this frequency of occurrence should not be disregard [15,16]. As a rule, the endoscopic pictures showing most predominant appearance was used for the assessment in this study, we could not detect the specific micro vascular patterns of well differentiated adenocarcinomas, which might be seen in the tiny area of these lesions. All four lesions that have well differentiated adenocarcinoma components in dense vascular pattern were 5 mm or larger in size (data not shown). Therefore, intensive follow up, polypectomy, or endoscopic mucosal resection may be recommended for such size of gastric protruding/or polypoid lesions with dense vascular patterns.

## Conclusions

In conclusion, we have shown that the magnifying NBI endoscopic appearances, especially the micro vascular patterns well correlates with histopathology of gastric protruding/or polypoid lesions, and provides useful information for discriminating GN from FGP and HP. This is the first investigating the potential usefulness of magnifying NBI endoscopy in diagnosing gastric protruding/or polypoid lesions. This finding will lead us to further longitudinal investigation of magnifying NBI endoscopy to know its clinical utility in the gastric lesions.

## Abbreviations

NBI: Narrow band imaging; *H. pylori: Helicobacter pylori; *FGP: Fundic grand polyp; HP: Hyper plastic polyp; GN: Gastric neoplasia.

## Competing interests

The authors declare that they have no competing interests.

## Authors' contributions

T O collected and analyzed data, Y K performed upper gastroscopy, collected and analyzed data, T T performed upper gastroscopy collected and analyzed data, and wrote manuscript. T S, M N, M O, and Y N performed upper gastroscopy and advised about manuscript. J Y, D Y, T I, N M, T K, H F, M N, M I, T A, I H advised about data analyzing and manuscript. All authors read and approved the final manuscript.

## Pre-publication history

The pre-publication history for this paper can be accessed here:

http://www.biomedcentral.com/1471-230X/12/17/prepub

## References

[B1] Gono K Obi T Yamaguchi M Appearance of enhanced tissue features in narrow-band endoscopic imaging J Biomed Opt 2004 9 568 577 10.1117/1.1695563 15189095

[B2] Goda K Tajiri H Ikegami M Urashima M Nakayoshi T Kaise M Usefulness of magnifying endoscopy with narrow band imaging for the detection of specialized intestinal metaplasia in columnar-lined esophagus and Barrett's adenocarcinoma Gastrointest Endosc 2007 65 36 46 10.1016/j.gie.2006.03.938 17185078

[B3] Sharma P Bansal A Mathur S The utility of a novel narrow band imaging endoscopy system in patients with Barrett's esophagus Gastrointest Endosc 2006 64 167 175 10.1016/j.gie.2005.10.044 16860063

[B4] Shibuya K Hoshino H Chiyo M High magnification bronchovideoscopy combined with narrow band imaging could detect capillary loops of angiogenic squamous dysplasia in heavy smokers at high risk for lung cancer Thorax 2003 58 989 995 10.1136/thorax.58.11.989 14586056 PMC1746520

[B5] Machida H Sano Y Hamamoto Y Narrow-band imaging in the diagnosis of colorectal mucosal lesions: a pilot study Endoscopy 2004 36 1094 1098 10.1055/s-2004-826040 15578301

[B6] Uedo N Ishihara R Iishi H A new method of diagnosing gastric intestinal metaplasia: narrow-band imaging with magnifying endoscopy Endoscopy 2006 38 819 824 10.1055/s-2006-944632 17001572

[B7] Bansal A Ulusarac O Mathur S Sharma P Correlation between narrow band imaging and nonneoplastic gastric pathology: a pilot feasibility trial Gastrointest Endosc 2008 67 210 216 10.1016/j.gie.2007.06.009 18226682

[B8] Tahara T Shibata T Nakamura M Gastric mucosal pattern by using magnifying narrow-band imaging endoscopy clearly distinguishes histological and serological severity of chronic gastritis Gastrointest Endosc 2009 70 246 253 10.1016/j.gie.2008.11.046 19386303

[B9] Nakayoshi T Tajiri H Matsuda K Magnifying endoscopy combined with narrow band imaging system for early gastric cancer: correlation of vascular pattern with histopathology (including video) Endoscopy 2004 36 1080 1084 10.1055/s-2004-825961 15578298

[B10] Yao K Iwashita A Tanabe H White opaque substance within superficial elevated gastric neoplasia as visualized by magnification endoscopy with narrow-band imaging: a new optical sign for differentiating between adenoma and carcinoma Gastrointest Endosc 2008 68 574 580 10.1016/j.gie.2008.04.011 18656862

[B11] Yamada T Ichikawa H X-ray diagnosis of elevated lesions of the stomach Radiology 1974 110 79 83 4808543 10.1148/110.1.79

[B12] Oberhuber G Stolte M Gastric polyps: an update of their pathology and biological significance Virchows Arch 2000 437 581 590 10.1007/s004280000330 11193468

[B13] Jain R Chetty R Gastric hyperplastic polyps: a review Dig Dis Sci 2009 54 1839 1846 10.1007/s10620-008-0572-8 19037727

[B14] Hattori T Morphological range of hyperplastic polyps and carcinomas arising in hyperplastic polyps of the stomach J Clin Pathol 1985 38 622 630 10.1136/jcp.38.6.622 4008664 PMC499259

[B15] Daibo M Itabashi M Hirota T Malignant transformation of gastric hyperplastic polyps Am J Gastroenterol 1987 82 1016 1025 3661508

[B16] Stolte M Sticht T Eidt S Ebert D Finkenzeller G Frequency, location, and age and sex distribution of various types of gastric polyp Endoscopy 1994 26 659 665 10.1055/s-2007-1009061 7859674

